# Wearable Vibration Sensor for Measuring the Wing Flapping of Insects

**DOI:** 10.3390/s21020593

**Published:** 2021-01-15

**Authors:** Ryota Yanagisawa, Shunsuke Shigaki, Kotaro Yasui, Dai Owaki, Yasuhiro Sugimoto, Akio Ishiguro, Masahiro Shimizu

**Affiliations:** 1 Department of Systems Science, Osaka University, 1-2 Machikaneyama-cho, Toyonaka, Osaka 560-0043, Japan; yanagisawa.ryota@arl.sys.es.osaka-u.ac.jp; 2 Department of System Innovation, Osaka University, 1-2 Machikaneyama-cho, Toyonaka, Osaka 560-0043, Japan; shimizu@sys.es.osaka-u.ac.jp; 3 Frontier Research Institute for Interdisciplinary Sciences, Tohoku University, 6-3 Aramaki aza Aoba, Aoba-ku, Sendai 980-8578, Japan; k.yasui@riec.tohoku.ac.jp; 4 Research Institute of Electrical Communication, Tohoku University, 2-1-1 Katahira, Aoba-ku, Sendai 980-8577, Japan; ishiguro@riec.tohoku.ac.jp; 5 Department of Robotics, Tohoku University, 6-6-01 Aoba, Aramaki, Aoba-ku, Sendai 980-8579, Japan; owaki@tohoku.ac.jp; 6 Department of Mechanical Engineering, Osaka University, 2-1 Yamadaoka, Suita, Osaka 565-0871, Japan; yas@mech.eng.osaka-u.ac.jp

**Keywords:** wearable vibration sensor, insect, wing flapping

## Abstract

In this study, we fabricated a novel wearable vibration sensor for insects and measured their wing flapping. An analysis of insect wing deformation in relation to changes in the environment plays an important role in understanding the underlying mechanism enabling insects to dynamically interact with their surrounding environment. It is common to use a high-speed camera to measure the wing flapping; however, it is difficult to analyze the feedback mechanism caused by the environmental changes caused by the flapping because this method applies an indirect measurement. Therefore, we propose the fabrication of a novel film sensor that is capable of measuring the changes in the wingbeat frequency of an insect. This novel sensor is composed of flat silver particles admixed with a silicone polymer, which changes the value of the resistor when a bending deformation occurs. As a result of attaching this sensor to the wings of a moth and a dragonfly and measuring the flapping of the wings, we were able to measure the frequency of the flapping with high accuracy. In addition, as a result of simultaneously measuring the relationship between the behavior of a moth during its search for an odor source and its wing flapping, it became clear that the frequency of the flapping changed depending on the frequency of the odor reception. From this result, a wearable film sensor for an insect that can measure the displacement of the body during a particular behavior was fabricated.

## 1. Introduction

Animals survive by adapting to the complexity of their environment. In particular, insects with a much smaller neural system than higher vertebrates are able to behave adaptively in complex environments. Some of the factors allowing for adaptation are the specific mechanisms embedded in an insect’s body that allow it to successfully interact with its environment. For this reason, research on developing a system with an embedded physicality that imitates that of an insect has been actively conducted [1]. In the case of flying insects with wings, in particular, it is possible to significantly change the surrounding environment by dynamically changing the way the wings flap. Flapping is an important factor not only because it has great mobility and flight stability that cannot be imitated by an unmanned aerial vehicle powered by a propeller, but also because it has the effect of drawing odorous substances in the direction of the insect and advantageously advancing its search. In recent years, insect behavior control mechanisms have been modeled, and micro-air vehicles with insect-like motion have been developed [2]. Moreover, the intake mechanism of an insect has been implemented in a search robot [3]. It is important to acquire the relationship between wing flapping and behavior to understand the interaction of flapping with the environment. Therefore, various measurement methods have been proposed in previous studies. There are three methods applied: (1) indirectly estimating the dynamics of the flapping of the wings from the posture information of the insect, (2) measuring the vibration of the thorax of the insect, and (3) measuring the flapping using an external observation system. In Method (1), flying insects are tethered in a flight mill or flight simulator, and flapping is calculated from the changes in the measured attitude [4,5,6]. In Method (2), to measure the force applied during the wing flapping of the insect, MEMS force sensors [7,8], torque sensors [9], and strain gauges [10] are attached to the thorax of the insect to measure the flapping. In Method (3), high-speed cameras are commonly used to track insect movements such as the wing deformation of both tethered and untethered insects [11,12,13,14,15]. To increase the available capture volume, cable-driven robots [16] and three-dimensional motion capture systems [17] have been developed along with lasers and optoelectronic devices used to capture insect movements in larger areas [18,19,20,21]. The advantages of these methods are useful for an analysis of the kinematics in the movement of the wings, but it is difficult to measure the closed-loop feedback mechanism for a behavior modulation caused by environmental changes from the flapping of the body’s wings because the wings themselves are not directly measured. To measure these relationships, it is necessary to determine the relationship between the behavioral output and wing dynamics at the same time.

Therefore, in this study, we constructed a novel wearable sensor that can be attached to the wings of the insects to track their behavior without using a large-scale experimental device. This enables us to measure the behavior of the wing itself, as well as the behavior of the wing, which enables us to observe the feedback system between the wing flapping and behavior. In previous studies [22,23,24,25,26], a wearable flexible sensor for humans with a relatively large sensor was studied, although there are almost no examples of attaching a sensor to an insect itself for measurement purposes. Hence, we apply conventional manufacturing of soft sensors in constructing a novel vibration sensor applicable to the insect scale. In addition, we investigate whether the fabricated vibration sensor can be attached to wings of different shapes to measure the flapping. Furthermore, we analyze the relationship between the behavior and flapping by measuring the flapping of insects during their behavior.

## 2. Problem Statement

In this study, we fabricate a wearable sensor for an insect. Specifically, the fabricated sensor is attached to the wing of an insect, and it can measure the flapping behavior. To achieve the research objectives,

- a wearable vibration sensor for an insect is developed,
- the flapping of the insect wings is measured with a vibration sensor, and
- changes in flapping during the insect’s behavior are analyzed.

To fabricate a flexible sensor, the method of kneading a conductive material into a soft material such as PDMS (polydimethylsiloxane) was adopted [25,26]. In this research, to create an extremely thin and flexible sensor, flat silver particles were mixed with a silicon polymer to form a thin sheet. To verify the basic performance of the fabricated sensor, we first measured and evaluated the sensor response to known vibrations. Next, we attached a sensor to the actual wing of an insect, measured how the sensor responded to highly unpredictable vibrations, and evaluated the results. At this time, measurements were taken on two types of insects with different wing morphologies. Finally, we carried out an experiment to measure changes in flapping during the insect’s behavior using this sensor and analyzed their relationship.

## 3. Development of the Wearable Vibration Sensor

### 3.1. Fabrication of the Silver Leaf Powder-Silicon Vibration Sensor

**Figure 1:**
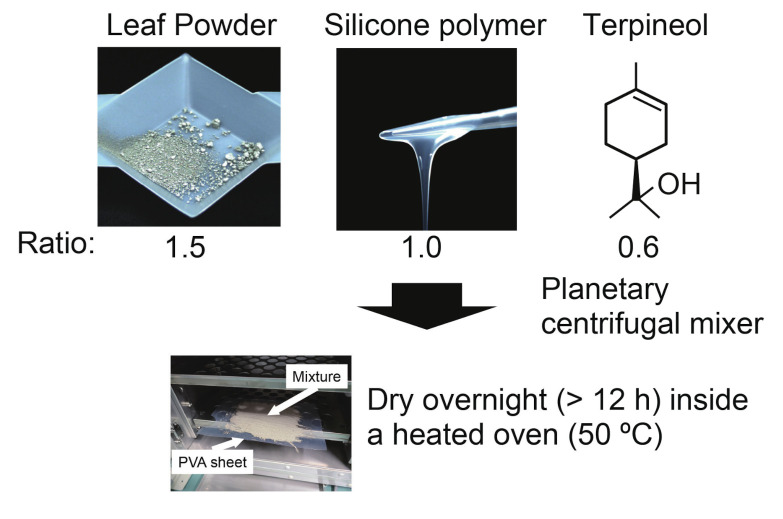
Sensor fabrication procedure.

**Figure 2:**
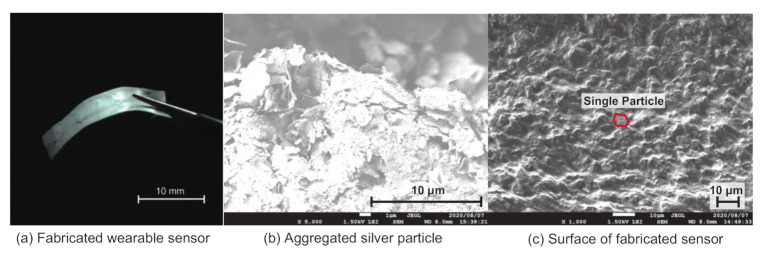
Images of the fabricated wearable sensor. (**a**) Overall image of the sensor (see Supplementary Material Video S1). (**b**) The flat silver particles are aggregated in the original form. (**c**) By admixing with the silicone polymer, the particles are spread evenly in the mixture. A typical image of a single silver particle is indicated with red lines.

A thin vibration sensor was fabricated through a three-step process as shown in Figure 1. First, a mixture composed of “LeafPowder” (47CE-5060, OIKE & Co. Ltd., Kyoto, Japan), which is made of flat silver particles, silicone polymer (Dragon Skin 10 Fast, Smooth-On, Inc., Macungie, PA, USA), and terpineol, was mixed at a weight ratio of 1.5:1:0.6 and thoroughly mixed using a planetary centrifugal mixer (AR-100, Thinky Co., Osaka, Japan). In the next step of the fabrication procedure, the previously prepared mixture was spread evenly on a polyvinyl alcohol sheet and dried overnight (>12 h) in a heated oven set to 50 ${}^{\circ }$C. This enabled most of the solvent to evaporate and helped solidify the silicon polymer. Finally, the fabricated batch sheet of the sensor was carefully released from the polyvinyl alcohol sheet and cut according to the intended use. For our study with insect wings, we employed a sensor with a length of 10 mm and a width of 5 mm, as shown in Figure 2a. The weight of the sensor at this time was about 12 mg. There are two advantages to using terpineol as a solvent. First, it increases the uniformity of the mixture subsequent to the mixing process. This results in an even resistor value throughout the sensor, owing to the dispersion of silver particles, which are aggregated in the original form. Second, it increases the ease of application when thinning out the paste, further reducing the thickness of the fabricated sensor to approximately 0.25 mm. For the purpose of our study of detecting the wing movement of the insect, the sensor, which had a thickness of 0.25 mm, was sufficiently thin and light to allow the insect to flap its wings.

### 3.2. Property Evaluation of the Thin Vibration Sensor

To analyze the material properties of the fabricated sensor, two types of analysis were conducted.

If we assume that the solvent is vaporized during the burning process, we can determine from the weight of the remaining ashes that the ratio between the silicon monomer (C${}_{2}$H${}_{6}$OSi) and silver particles (Ag) is roughly 1:0.93 (±0.3). When converted into weight percentage, the weight of silver particles accounts for 57.50% of the overall weight of the sensor. Given that the selected weight ratio for the silver particles was 1:1.5, or 60 % of the overall sensor weight, our results indicate that, through this fabrication protocol, the uniformity of the silver particle density is retained regardless of where we cut the sensor from the batch sheet.

In the second part of the analysis, scanning electron microscopy (JSM-7500F, JEOL Ltd., Tokyo, Japan) was used to evaluate the relative relationship between each silver particle within the silicon polymer. Figure 2b shows the microscopic image of the silver powder used in the experiment. Each bulk of silver powder was composed of aggregated flat silver flakes with an average length of 3–4 μm. By admixing the silicon polymer with the silver particles, as shown in Figure 2c, the particles were dispersed evenly in three-dimensional space. This results in a complicated network of flat silver particles, enabling the electrical current to flow through the sensor regardless of the bending direction.

### 3.3. Experimental Evaluation of the Sensor Response

**Figure 3:**
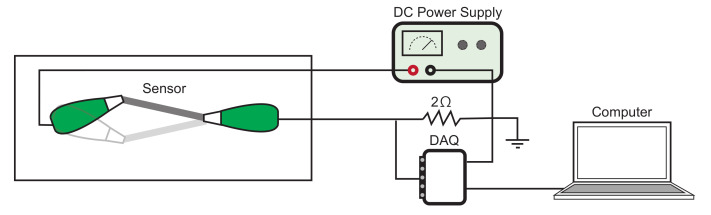
Schematic of the setup used to evaluate the frequency response of the fabricated sensor.

To evaluate the sensor response to a bending deformation, a testing device composed of a servo motor and a 3D printed base was used to emulate the flapping behavior of an insect wing, as shown in Figure 3. Both ends of the sensor were connected to the circuit using a pair of alligator clips with one end fixed to the base plate and the other end attached to a servo motor. Using an Arduino Uno, the motor was programmed to change one degree per 10 ms for three different angles (20, 40, and 60 degrees). This means that for each of the angles tested, the velocity remained constant throughout the testing period. To measure the sensor response to angle displacement, the sensors were first applied with a constant voltage through a DC power supply and then processed through a voltage divider before being sampled with a data acquisition interface (DAQ-6218, National Instruments Co., Austin, TX, USA) at a rate of 1 kHz. Once the data were collected, the acquired data were detrended by removing the straight-line fit using MATLAB. The frequency was derived by sampling 1 s worth of data, or 1000 data points, and applying FFT (fast Fourier transform) to the time domain information.

Table 1 shows the results gained from a sensor evaluation using the aforementioned testing device. The rows in the table show the displacement angle of the servo motor, the frequency analysis results of the signal input to the servo, the frequency analysis results of the sensor signal, and the difference between the true frequency and the frequency of the sensor, respectively. Because the experiment was conducted three times for each angle, the mean and standard deviation are shown in the table. The results show that our sensor can detect the displacement frequency for different angle deformations. This suggests that the fabricated sensor has a function for detecting vibrations.

## 4. Measurement of Insect Wing Flapping with a Wearable Vibration Sensor

### 4.1. Experimental Conditions

**Figure 4:**
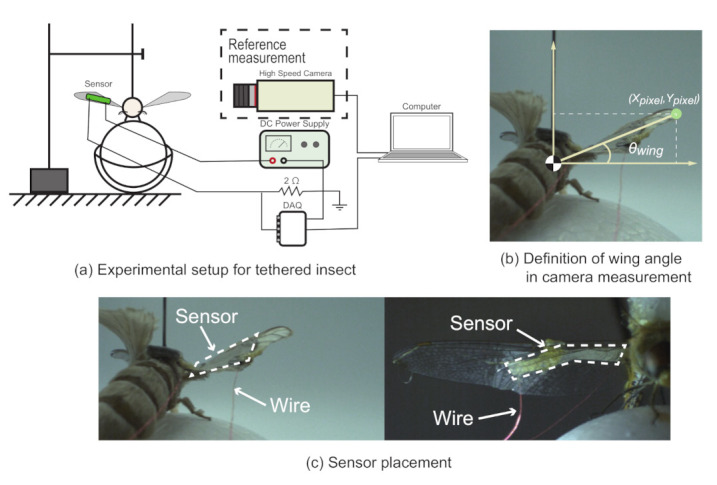
Experimental setup of tethered insects. A schematic of the testing environment is shown in (**a**), and the wing angle used for the experiment is shown in (**b**). A typical image of the sensor placement on a silk moth and a dragonfly is shown in (**c**) (see Supplementary Material Video S2).

In this section, we experimentally verify whether a wearable sensor can measure the wing flapping of insects. First, we investigate the effect of attaching the sensor to the wing of an insect by measuring the change in flapping frequency and flapping angle using a high-speed camera. Next, we check whether it is possible to actually measure the flapping of the wing of the insect from the output values of the wearable sensor. In these two experiments, we employed different individuals. The specific experimental conditions are described below. As shown in Figure 4, a testing setup housed within a photographing box was used to evaluate the wing movement of the insects and their corresponding sensor output.

The setup was evenly lit from above, using LED strips, and the wing movement was captured using a high-speed camera (HAS-U1, DITECT Co. Ltd, Osaka, Japan), set to a frame rate of 800 fps. The insect was tethered by first applying a thin coat of quick-drying adhesive on the back of the insect and then attaching it to double-sided tape placed at the end of an L-shaped metal rod, which was held in place by an XYZ linear translation stage. As shown in Figure 4, a styrofoam ball was placed at the bottom of the insect with the linear translation stage adjusted such that the tethered insect could freely move the styrofoam ball in any direction using its legs. To measure the flapping behavior, the fabricated sensor with a length of 10 mm was attached to the insect wing with a thin coat of a quick-drying adhesive. Both ends of the sensor were folded inward with thin wires enclosed using a glue stick.

The sensor measurement setup was the same as described in the previous section. Sensor measurements were conducted by applying a constant voltage to one end of the sensor using a DC power supply. The other end of the sensor was processed through a voltage divider of 2 Ω before being sampled through the data acquisition system, which was connected to a computer. Sensor signals were sampled using the data acquisition system at a rate of 1 kHz. As with the sensor response experiment described in the previous section, noise and DC component removal were also conducted using the wavelet transform during the experiment using actual insects. The wing motion from the high-speed camera video footage was tracked using the object tracking feature in KINOVEA [27]. KINOVEA is an open source motion analysis software. The wing angle was derived by relocating the coordinate origin to the center of the insect body and calculating the inverse tangent of the wingtip position ($X_{\mathrm{pixel}}$, $Y_{\mathrm{pixel}}$) using the following equation:

$$\theta_{\mathrm{wing}}=\arctan\frac{Y_{\mathrm{pixel}}}{X_{\mathrm{pixel}}}.$$

Employing the experimental setup for the tethered insect, we measured the flapping behaviors of a silk moth (*Bombyx mori*) and a dragonfly (*Sympetrum darwinianum*). The wing weights of the silk moth and the dragonfly were approximately 1.5 mg (measured with an electronic balance) and 3 mg [28], respectively. Both of these insects are known to have inherent differences in the motivation behind their wing flapping behavior, with different wing morphologies to meet their needs. To accommodate their differences, two types of procedures were selected to initiate the wing movement. For a silk moth, an airflow containing a sex pheromone (bombykol [29]) was diffused toward the antenna, thus activating its wing flapping. However, in the case of a dragonfly, the styrofoam ball that was used to support the insect legs was removed to initiate wing movement during flight. This is a commonly reflexive behavior of flying winged insects.

### 4.2. Results

**Figure 5:**
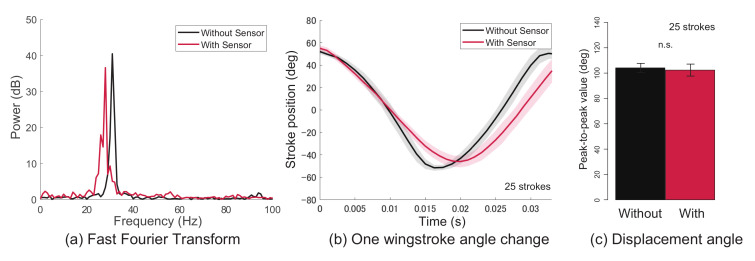
Evaluation of the effect of flapping when the sensor is attached to a silk moth. (**a**) Flapping frequency, (**b**) the flapping displacement angle change of one wingstroke, and (**c**) the peak-to-peak value of the flapping angle.

**Figure 6:**
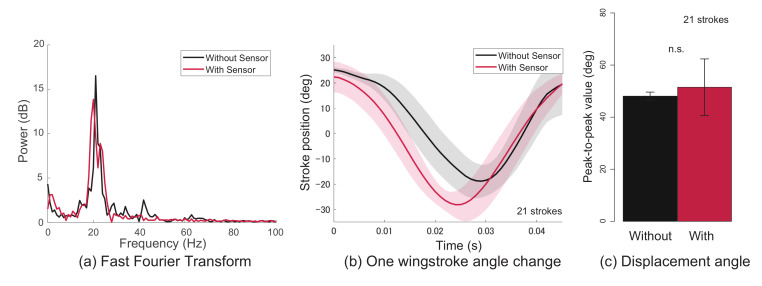
Evaluation of the effect of flapping when the sensor is attached to a dragonfly. (**a**) Flapping frequency, (**b**) the flapping displacement angle change of one wingstroke, and (**c**) the peak-to-peak value of the flapping angle.

First, the results of the flapping frequency and flapping displacement angle before and after mounting the sensor on the wing are shown. Figure 5 and Figure 6 show the results of a silk moth and dragonfly, respectively. Furthermore, (a)–(c) in the figures are the flapping frequency, the flapping displacement angle change of one wingstroke, and the peak-to-peak value of the flapping angle, respectively. The black line in the figures is the data before the sensor is attached, and the red line is the data after the sensor is attached. The data before and after mounting the sensor are the data obtained from the same wing of the same individual. From the results in Figure 5 and Figure 6, it was clarified that there was no big difference in the flapping frequency and flapping displacement angle change even when the sensor was attached. Additionally, as a result of testing the peak-to-peak value, there was no significant difference between before and after wearing the sensor (Wilcoxon rank sum test, p<0.05). From these results, it was clarified that the effect of flapping on the sensor was small. Next, the results of measuring the flapping with a wearable sensor are shown.

**Figure 7:**
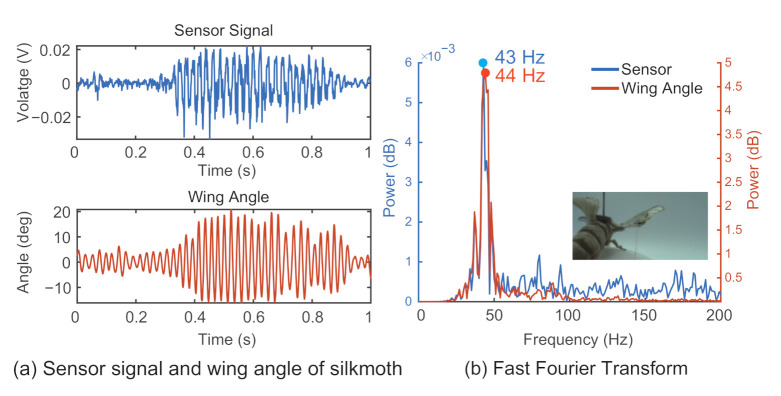
Sensor signal and wingbeat of a tethered silk moth. The results for the measured sensor signal and the corresponding wing angle are shown in (**a**). FFT and peak values are shown in (**b**).

**Figure 8:**
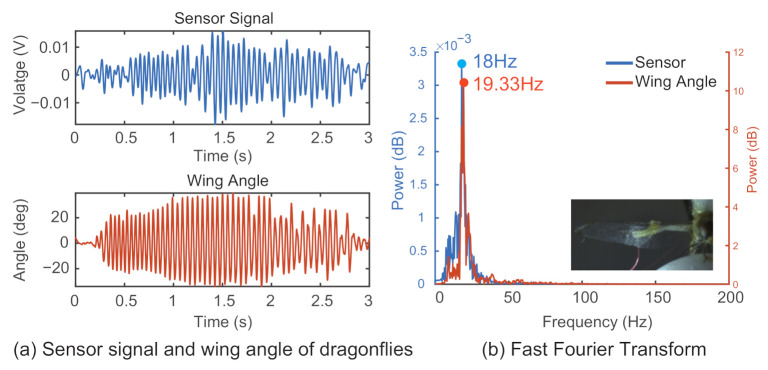
Sensor signal and wingbeat of a tethered dragonfly. The results of the measured sensor signal and the corresponding wing angle are shown in (**a**). FFT and peak values are shown in (**b**).

Typical results for the wing flapping behavior of the silk moth are shown in Figure 7a, and those of the dragonfly are shown in Figure 8a. To further analyze the relationship between the sensor signal and the insect wing flapping, FFT was used to convert the given time domain signal into frequency domain information. The FFT results for the silk moth are shown in Figure 7b, and the result for the dragonfly are shown in Figure 8b. In both cases, the lines in blue represent the data from the sensor signal, and the lines in orange represent the wingbeat retrieved from the high-speed camera footage.

A comparison between the measured sensor signal with the wingbeat derived from high-speed camera recordings was conducted by extracting the peak frequency values from the frequency domain information for both a silk moth and a dragonfly, as shown in Figure 7 and Figure 8, respectively. In Figure 7b, the peak frequency value for the sensor signal was 43 Hz, and that for the wingbeat was 44 Hz. In the case of Figure 8b, the peak point from the frequency domain for the sensor signal was 18 Hz, and that for the wingbeat was 19.33 Hz. Table 2 shows the results from the silk moth, and Table 3 shows the results from the dragonfly. The rows in the table show the results of the high-speed camera, the results of the sensor signal, and the difference between the high-speed camera frequency (true) and the frequency of the sensor. From this result, it is possible that their flapping was close to their natural form despite the attachment of the sensor because the flapping frequency of the silkworms is between 20 and 50 Hz [30] and the flapping frequency of dragonflies is between 15 and 30 Hz [31].

## 5. Analysis of Flapping Changes with Behavior

### 5.1. Experimental Conditions

One of the main advantages of our novel sensor is its ability to be incorporated into the existing testing environment to gain further insight into the relationship between the wing movement and other behaviors exhibited by an insect. To demonstrate the potential of our novel sensor, we focused on the relation between the odor search behavior of a silk moth and the corresponding wingbeat frequency for the duration of the search. The silk moth is known to realize an efficient search by intaking the surrounding odorous substances in its own direction by flapping its wings [32]. However, it is still unknown how much the flapping frequency changes during the search. Therefore, we clarify the relationship between the two by measuring the search behavior while obtaining the frequency of the flapping using the novel sensor.

Using a virtual reality system [33] designed to emulate the pheromone dispersion in an untethered environment for a tethered silk moth, we calculated the wingbeat frequency by measuring the output signal from the fabricated sensor attached to both wings. The virtual reality system used for the odor search experiment was composed of sensors that detect the direction of the insect movement and two solenoid valves attached to a pump stimulating the corresponding antenna with a pheromone (bombykol) based on a diffusion simulation in a virtual environment. In the initial state, the tethered silk moth was placed 300 mm away from the odor source in the horizontal direction. After initialization, a flow of pheromone was released from the odor source at a constant frequency (1 Hz) and dispersed as it traveled in-plane. When the insect came into contact with the pheromone in the virtual environment, the tethered insect was provided with a pheromone stimulation to the corresponding antenna. The simulation ended either when the silk moth reached the goal area (a 50 mm radius from the odor source) or when it reached the 5 min time limit. A measurement of the sensor values was conducted using the same setup as that applied for the tethered insect.

### 5.2. Experimental Results

**Figure 9:**
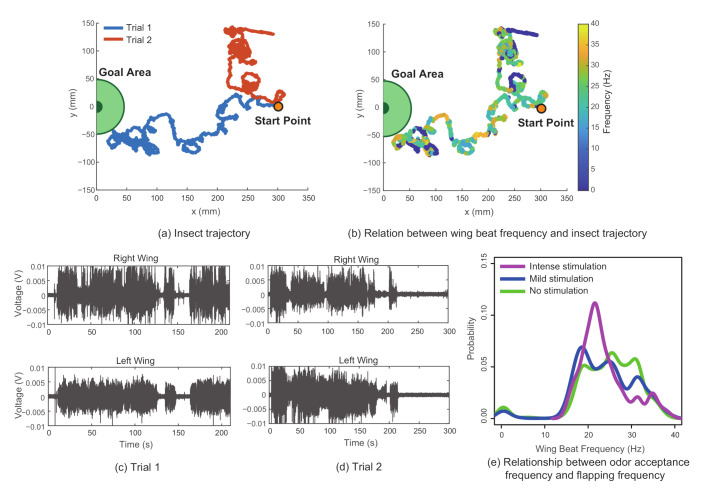
Results for the odor search behavior of a silk moth. The trajectory of insect movement during odor search behavior is shown in (**a**), and the corresponding wingbeat frequency is shown in (**b**). The measured sensor signals for both the right and left wings during Trials 1 and 2 are shown in (**c**,**d**), respectively. The probability of the wingbeat frequency for three different types of pheromone simulations is shown in (**e**), where an intense stimulation indicates stimulation frequencies greater than 1 Hz, a mild stimulation refers to a frequency between 0.5 and 1 Hz, and no stimulation indicates less than 0.5 Hz.

Figure 9a shows the insect trajectory for two different results. Trial 1 refers to the case in which the silk moth was able to locate the odor source, and Trial 2 refers to the case in which the silk moth failed to find the odor source. The search time for Trial 1 was 3.5 min. The measured sensor values for both cases are shown in Figure 9c,d, respectively. To derive the change in wingbeat frequency during the odor search behavior, we calculated the spectrogram for the right wing using the short-time Fourier transform. The frequency estimation of the sampled insect position was processed by interpolating the nearest frequency values for each sampled timestamp of the insect position, as shown in Figure 9b).

As stated earlier, the silk moth is known to actively flap its wings in order to accumulate the surrounding pheromones to their antenna and cancel out unwanted plumes from behind. This unique behavior in combination with three different walking patterns (zigzag, surge, and loop) triggered by pheromone stimulation is thought to contribute to their effective odor search mechanisms [34]. It is also known that odor source search behavior is regulated by the frequency of the odor-receptive stimulation [34,35]. It is quite possible for the frequency of the flapping to change depending on the frequency of the odor reception because walking has a high correlation with flapping [36,37]. Therefore, we analyzed the relationship between the frequency of the odor reception and the frequency of the flapping. The frequency of the odor reception was calculated based on the number of times the odor was presented to the silk moth. The result is shown in Figure 9e. The green, blue, and purple colors in Figure 9e indicate when a stimulus is not present (“No stimulation”, 0.5 <= f ([the amount of odor stimulus/s)), when the odor hits approximately once (“Mild stimulation”, 0.5 < f (the amount of odor stimulus/s) <= 1), and when the odor hits with high frequency (“Intense stimulation”, f (the amount of odor stimulus/s) > 1), respectively. The vertical axis in Figure 9e represents the probability of appearance, and the horizontal axis represents the frequency of the flapping. This figure suggests that silk moths do not always flap at the same frequency during exploratory behavior. The silk moth flapped at approximately 30 Hz when no odor stimulus was detected, and its distribution changed when it received an odor stimulus, in which it flapped at a frequency of less than 20 Hz. However, we found that when the odor was continuously received, the insect flapped strongly at a frequency of 20 Hz. The insect attempted to receive odor information by actively attracting the surrounding odor to itself by flapping at a relatively high frequency when an odor was not sensed. However, when odor information was received, it is thought that the disturbance of the surrounding airflow was suppressed by lowering the flapping frequency. In other words, it is hypothesized that the silk moth may change the body search mode of its flapping according to the amount of odor information.

## 6. Discussion

A comparison between the sensor signal and high-speed camera footage indicates that our novel vibration sensor is capable of detecting the wingbeat frequency for two insect wing types with relatively high precision, opening up the possibility of providing an alternative solution for measuring insect wing deformation. Although we were able to measure insect wings with different morphologies, one of the limitations of the current sensor is the frequency range that the sensor is capable of detecting. Insects such as bees with a higher beat frequency (>100 Hz) require a sensitive sensor with a well-balanced signal-to-noise ratio. Given that, in its current form, our sensor is capable of sensing a frequency range of up to 40 Hz, we expect sensors capable of detecting higher frequency ranges to be fabricated by utilizing silicone polymers with different material properties.

**Figure 10:**
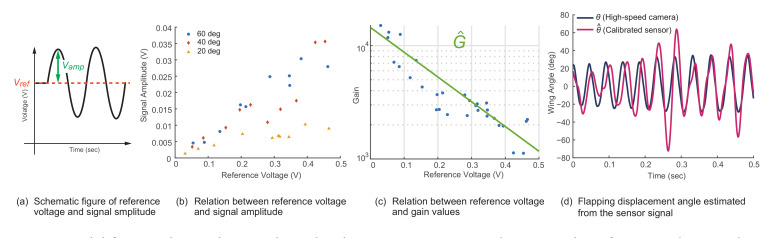
Model for translating the signal amplitude into insect wing angle. Using the reference voltage and signal amplitude defined in (**a**), their relationships are shown in (**b**), and the exponential trend line is shown in (**c**). Using the linear model, the wing angle trajectory of a dragonfly is compared with the calibrated sensor signal (**d**).

Beyond incorporating the sensor into existing setups, another interesting application is the possibility of estimating the wing angle of insects from a measured signal. From the sensor data obtained, we assumed that if we derive the gain values from the DC component of the signal, we can acquire a rough linear model that translates the sensor signals into the angle displacement. Herein, we define the average sensor values of the measured data as the reference voltage and the signal amplitude as the amplitude obtained from the sensor signal after denoising and detrending the original signal using the aforementioned multi-resolution analysis, as shown in Figure 10a.

Figure 10b shows the relationship between the reference voltage and the resulting signal amplitude for three different angle ranges of between 20 and 60 degrees. As indicated in the results with a displacement of 20 degrees, the signal amplitude reaches a steady state after exceeding a certain reference voltage. Given this result, we defined the gain values as below and plotted the relation between the gain values and the reference voltage in Figure 10c.

(1) $$\mathrm{Gain}=\frac{\theta_{\mathrm{Displacement}}}{2V_{\mathrm{Amp}}}$$

From the results obtained, we derived an exponential trend line, as shown in Figure 10c, that roughly translates the measured reference voltage into the gain values. The equation is expressed as follows:

(2) $$\hat{G}=15\times 10^{3}\exp^{-5V_{\mathrm{Ref}}}.$$

Using Equation (3), we can infer the angular displacement by multiplying the denoised and detrended sensor signal with the gain values derived from Equation (2). An example comparing the wing trajectory of a dragonfly with the results for the calibrated sensor is shown in Figure 10.

(3) $${\hat{\theta }}_{\mathrm{Displacement}}=2\hat{G}V_{\mathrm{Amp}}$$

Although there are limitations to the fitness of the estimated wing angle using the derived model, a Gaussian process regression can be used to further optimize the estimated output.

## 7. Conclusions

In this study, we fabricated a wearable sensor to measure the flapping of insect wings and measured the flapping of the wings of two species of insects with different wing morphologies.

Our novel vibration sensor was fabricated by mixing flat silver particles called “LeafPowder” with a silicone polymer. This sensor was attached to two types of insects (a silk moth and a dragonfly) with different wing morphologies, and an experiment was conducted to determine whether the flapping frequency could be measured. As a result, the flapping frequency calculated by the camera and the flapping frequency measured by the sensor mostly matched, and we succeeded in fabricating a wearable vibration sensor for an insect. In addition, using this vibration sensor, we acquired and analyzed the relationship between the odor source search behavior of the silk moth and the flapping frequency. As a result, it was suggested that the silk moth changes the flapping mode depending on the frequency of the odor reception.

Although the proposed sensor is good at detecting vibration, the accuracy of the displacement estimation is insufficient. However, because the signal-to-noise ratio can be improved by changing the content of the leaf powder and the type of silicone resin, we will continue to study these aspects in the future. We will also acquire the relationship between the movement trajectory of dragonflies in actual free flight and their wing flapping frequency and clarify a part of their movement mechanism.

## Figures and Tables

**Table 1:** Results of the frequency response to three different angle deformations given to the sensor. “Difference freq.” is the absolute value of the true frequency minus the sensor frequency.

Angle (deg)	True Freq. (Hz)	Sensor Freq. (Hz)	Difference Freq. (Hz)
20	2.5 ± 0.0	2.5 ± 0.0	0.0 ± 0.0
40	1.3 ± 0.0	1.0 ± 0.0	0.3 ± 0.0
60	0.83 ± 0.0	1.0 ± 0.0	0.17 ± 0.0

**Table 2:** Comparison between the frequency response of the sensor to the actual wingbeat frequency of silk moths obtained from high-speed camera footage. “Difference freq.” is the absolute value of the camera frequency minus the sensor frequency. “SM” stands for silk moth.

ID	Trial	True Freq. (Hz)	Sensor Freq. (Hz)	Difference Freq. (Hz)
	1	26	26	0
SM1	2	19	20	1
	3	18	18	0
	1	28	30	2
SM2	2	26	26	0
	3	18	21	3
	1	17	17	0
SM3	2	16	19	3
	3	17	18	1
	1	31	31	0
SM4	2	23	23	0
	3	23	23	0
	1	43	43	0
SM5	2	41	41	0
	3	40	40	0

**Table 3:** Comparison between the frequency response of the sensor to the actual wingbeat frequency of a dragonfly obtained from high-speed camera footage. “Difference freq.” is the absolute value of the camera frequency minus the sensor frequency.

Trial	True Freq. (Hz)	Sensor Freq. (Hz)	Difference freq. (Hz)
1	19	19	0
2	19	18	1
3	20	20	0
4	19	19	0

## Data Availability

The MATLAB code introduced in the paper can be found at https://sshigaki.jimdo.com/research/.

## Supplementary Materials

The following is available online at https://sshigaki.jimdo.com/research/: Video S1: Outline video of the sensor; Video S2: Experimental video of the silk moth and dragonfly wing flapping measurements.

## Abbreviations

The following abbreviations are used in this manuscript:

FFT	Fast Fourier transform
PDMS	Polydimethylsiloxane
